# Exploring factors of diagnostic delay for patients with bipolar disorder: a population-based cohort study

**DOI:** 10.1186/s12888-020-2483-y

**Published:** 2020-02-19

**Authors:** Ágnes Lublóy, Judit Lilla Keresztúri, Attila Németh, Péter Mihalicza

**Affiliations:** 1 Department of Finance and Accounting, Stockholm School of Economics in Riga, Strēlnieku iela 4a, Rīga, LV-1010 Latvia; 2 Department of Finance, Corvinus University of Budapest, Fővám tér 8, Budapest, 1093 Hungary; 3 Directorate, National Institute for Psychiatry and Addictions, Lehel utca 59-61, Budapest, 1135 Hungary; 4 Doctoral School, Semmelweis University, Üllői út 26, Budapest, 1085 Hungary

**Keywords:** Bipolar disorder, Diagnostic delay; Cox proportional hazards model, Patient pathway, Hungary, Mental health services

## Abstract

**Background:**

Bipolar disorder if untreated, has severe consequences: severe role impairment, higher health care costs, mortality and morbidity. Although effective treatment is available, the delay in diagnosis might be as long as 10–15 years. In this study, we aim at documenting the length of the diagnostic delay in Hungary and identifying factors associated with it.

**Methods:**

Kaplan-Meier survival analysis and Cox proportional hazards model was employed to examine factors associated with the time to diagnosis of bipolar disorder measured from the date of the first presentation to any specialist mental healthcare institution. We investigated three types of factors associated with delays to diagnosis: demographic characteristics, clinical predictors and patient pathways (temporal sequence of key clinical milestones). Administrative data were retrieved from specialist care; the population-based cohort includes 8935 patients from Hungary.

**Results:**

In the sample, diagnostic delay was 6.46 years on average. The mean age of patients at the time of the first bipolar diagnosis was 43.59 years. 11.85% of patients were diagnosed with bipolar disorder without any delay, and slightly more than one-third of the patients (35.10%) were never hospitalized with mental health problems. 88.80% of the patients contacted psychiatric care for the first time in outpatient settings, while 11% in inpatient care. Diagnostic delay was shorter, if patients were diagnosed with bipolar disorder by non-specialist mental health professionals before. In contrast, diagnoses of many psychiatric disorders received after the first contact were coupled with a delayed bipolar diagnosis. We found empirical evidence that in both outpatient and inpatient care prior diagnoses of schizophrenia, unipolar depression without psychotic symptoms, and several disorders of adult personality were associated with increased diagnostic delay. Patient pathways played an important role as well: the hazard of delayed diagnosis increased if patients consulted mental healthcare specialists in outpatient care first or they were hospitalized.

**Conclusions:**

We systematically described and analysed the diagnosis of bipolar patients in Hungary controlling for possible confounders. Our focus was more on clinical variables as opposed to factors controllable by policy-makers. To formulate policy-relevant recommendations, a more detailed analysis of care pathways and continuity is needed.

## Background

Bipolar disorder is a chronic mental disorder that causes periods of depression and periods of abnormally elevated mood, such as mania or hypomania. The dramatic episodes of high and low moods do not follow a set pattern, it may vary patient by patient. In between the dramatic episodes patients usually feel normal. The lifetime prevalence of bipolar disorder is estimated to be approximately 1 % both in Europe and in the US [1]. Some studies, however, report a much larger prevalence rate: in five studies reviewed in [1] the prevalence rate estimates range from 2.6 to 6%. Mental disorders, including depression, bipolar affective disorder, and schizophrenia are considered among the leading causes of disability worldwide [2]; bipolar disorder alone is documented to be the 12th leading cause of disability worldwide [3].

For mental health professionals, it is difficult to distinguish bipolar disorder from other mental disorders. Patients are often misdiagnosed at the initial presentation at mental healthcare institutions; they mostly receive the initial diagnosis of unipolar depression, schizophrenia or substance-induced psychotic disorder [4–6]. The delay in diagnosis might be as long as 10–15 years [5, 7–9]. Late diagnosis of bipolar disorder has severe consequences. Bipolar disorder, if untreated or treated with antidepressants, is coupled with higher rates of self-harm and suicide [10, 11]. Late diagnosis contributes to various forms of substance abuse [11, 12]. Untreated bipolar disorder causes severe role impairment, like loss of ability to work and difficulties in maintaining personal relationships with family members, friends and colleagues [13]. It is also coupled with high direct and indirect health care costs, because of, among others, long hospitalizations [14], and high social costs [15].

Given the severe consequences of delays to diagnosis, this study aims at identifying factors associated with diagnostic delay for patients with bipolar disorder. In this study, we analyze administrative data from specialist care for a large population-based Hungarian cohort. We develop a strict definition of receiving the first bipolar disorder diagnosis: only diagnoses made at a specialist mental health provider are considered. First, we assess whether the time to diagnosis of bipolar disorder measured from the date of the first presentation to any specialist mental healthcare institution is substantial. Second, we investigate three types of factors associated with delays to diagnosis: demographic characteristics of patients, clinical predictors and patient pathways.

Regarding *demographic characteristics,* patient age at the time of the first presentation to mental healthcare institutions might influence diagnostic delay: while studying the incidence and distribution of first-episode mania by age, Kennedy et al. [16] identified early and late-onset subgroups. They found that the incidence of mania generally peaks in early adult life and has a smaller peak between 40 to 55 years-of-age. Moreover, Berk et al. [17] argue that for early-onset groups, the pattern of symptoms might not overlap with the criterion-based ICD diagnostic classification which may result in diagnostic and treatment delays. *Clinical predictors* capture whether the probability of receiving the first bipolar diagnosis late is associated with previous diagnoses received in specialist mental healthcare. It is reasonable to hypothesize that the time to diagnosis of bipolar disorder measured from the date of the first presentation to any specialist mental healthcare institution is longer, if symptoms indicative of underlying bipolar disorder have been attributed to another mental illness or comorbid alcohol and substance misuse [18–20]. Physicians might be reluctant to change these previous diagnoses, which in turn increases the probability of being diagnosed late with bipolar disorder for the first time. *Patient pathway variables* assess whether the temporal sequence of key clinical milestones is associated with delays to diagnosis. These clinical milestones, among others, include the point of first contact with mental healthcare service and the place of the first bipolar diagnosis (outpatients vs inpatient care), and whether the patient was hospitalized prior to the date of the first bipolar diagnosis or not. A better understanding of factors associated with diagnostic delay for patients with bipolar disorder might help developing strategies to reduce it.

This research is a first attempt to systematically describe and analyse in a nationwide cohort the lag in the diagnosis of bipolar patients presenting to specialist care while controlling for possible confounders. This study makes three contributions to the literature on the diagnostic delay in patients with bipolar disorder. First, our research is based on administrative data which has the advantage of the large sample size. The population-based cohort includes almost 9000 patients with bipolar disorder; the sample is more than six times larger than in [20], the research which is the most similar to the current study. The second important contribution of the study is the inclusion of patient pathway variables assessing whether the temporal sequence of key clinical milestones is associated with delays to diagnosis. To our knowledge, no previous research has tested empirically the relationship between the diagnostic delay in patients with bipolar disorder and patient pathways. Third, although the time from the first psychiatric diagnosis to the bipolar diagnosis has been estimated from nationwide registries in some countries, such as Sweden and Denmark [7, 21], no similar estimates are available for Central and Eastern Europe. In Central and Eastern Europe, the socio-economic status of patients is lower on average than in the most developed countries in the world and the health care systems are facing different challenges, which does not allow the the generalization of previous findings.

## Methods

### Sample

Anonymized data were retrieved from the administrative financing database collected by the National Health Insurance Fund Administration of Hungary (NHIFA) and maintained by the National Healthcare Service Centre of Hungary. Usage has been approved by the National Healthcare Services Centre of Hungary (AEEK/4538/2016).

All Hungarian residents aged between 18 and 65 years were eligible for entering the study. Patients were included in the sample if they were diagnosed with bipolar disorder for the first time from 1 January 2015 to 31 December 2016 in acute inpatient or outpatient care. By definition, patients were diagnosed with bipolar disorder, if one of the following criteria were met:

- ▪ The patient was assigned with the ICD-10 diagnostic code of F31 as a principal diagnosis at a psychiatric unit, either outpatient or inpatient.
- ▪ The patient was assigned with the ICD-10 diagnostic code of F31 as secondary diagnosis at addictology inpatient units. (Although bipolar disorder may meet the criteria for principal diagnosis, in addictology only addiction-related ICD-10 codes can be used as principal diagnosis.)
- ▪ The patient was assigned with the ICD-10 diagnostic code of F31 as secondary diagnosis with principal diagnosis of F20 provided at psychiatric inpatient units. (When the diagnostic code of F20 is sequenced first, professionals may prescribe both antipsychotics and mood stabilizers.)

In Hungary, general practitioners theoretically act as first points of contact for patients, and as gatekeepers for secondary care. With mental health problems patients typically consult their general practitioner first who may then initiate a referral to specialists [22]. Alternatively, patients may self-diagnose their condition and visit psychiatric professionals without any referral. Patients entered the study if they were diagnosed with bipolar disorder at specialist mental health services, either inpatient or outpatient, thus the diagnoses made by primary care providers or other healthcare professionals were not considered.

This strict definition ensures correct and trustworthy identification of bipolar disorder as less reliable diagnosis made by non-specialist mental health professionals is ruled out. At the same time, the definition guarantees that only patients who were channelled to specialist mental healthcare services are considered, making attribution of outcomes to the mental health system more legit. Please note that this definition implicitly assumes that patients at the time of the first presentation to any specialist mental healthcare institution have already experienced the onset of bipolar disorder. This implicit assumption is imposed by similar studies using claims data [20]. Acknowledging the age at onset of bipolar disorder is estimated to be 20–25 years [8, 23, 24], while the mean age of patients at the time of the first presentation to any specialist mental healthcare institution in the sample is 37.34, it is reasonable to assume that patients have already experienced the onset of bipolar disorder at the time of the first presentation.

### Outcome variable: diagnostic delay

The outcome measure is the time to the first diagnosis of bipolar disorder measured from the date of the first presentation to any specialist mental healthcare institution. The first presentation is captured by an F00–99.xx ICD-10 diagnosis given at psychiatric/addictology outpatient or inpatient settings. The time to diagnosis measured this way articulates the delay of bipolar disorder diagnosis after patients already contacted specialist mental healthcare. This definition of diagnostic delay is in agreement with the one proposed for patients with psychogenic nonepileptic seizure [25], for children with attention-deficit/hyperactivity disorder [26], and for patients with bipolar disorder [20].

### Demographics and clinical predictors

Electronic health records collected and maintained by the National Health Insurance Fund Administration of Hungary were used to extract the predictor variables. In order to track past medical history, we spanned a time period from 1 January 2004 to 31 December 2016, covering 13 years of medical history as a maximum. Demographic predictors included patients’ gender and age in the year of the first bipolar diagnosis. For clinical predictors, diagnoses assigned prior to the date of the first bipolar diagnosis were considered only. Clinical predictor variables entered the regression equation separately for inpatient and outpatient care, and they included the following variables: alcohol misuse/dependence (ICD-10 F10.x), illicit drug misuse/dependence (ICD-10 F11-F19.x), schizophrenia and related disorders (ICD-10 F20-F29.x), anxiety disorder (ICD-10 F40-F43.x), specific, mixed and other personality disorders other than borderline personality disorder, and enduring personality changes (ICD-10 F60-F62.x excluding F60.3), borderline personality disorder (ICD-10 F60.3), psychotic depression (ICD-10 F32.3/F33.2), unipolar depression without psychotic symptoms (ICD-10 F32.x/F33.x excluding F32.3/F33.2), mood affective disorders other than the ones listed above (ICD-10 F30-F39.x excluding F31-F33.x), organic, including symptomatic, mental disorders (ICD-10 F00-F09.x), neurotic, stress-related and somatoform disorders (ICD-10 F44-F48.x), behavioural syndromes associated with physiological disturbances and physical factors (ICD-10 F50–59.x), disorders of adult personality and behaviour other than the ones listed above (ICD F63-F69.x), mental and behavioural disorders other than the ones listed above (ICD-10 F70-F99.x), and intentional self-harm (ICD-10 X60–84.x). We counted the number of times the patient was assigned the above diagnoses while consulting specialist mental healthcare professionals in outpatient care or being hospitalized. We also counted the number of incidents of being hospitalized owing to poisoning and toxic substances chiefly nonmedicinal as to source (ICD-10 T36-T65.x). Three binary variables were defined as well. The first binary variable captured whether the first bipolar diagnosis was given in outpatient or inpatient care; the second indicated whether the patient’s first contact with mental healthcare service was in outpatient or inpatient care. The third binary variable captured whether the patient was hospitalized prior to the date of the first bipolar diagnosis or not. Finally, one variable measured how many times did the patient receive an F31 diagnosis that is not in line with the three criteria, listed earlier, for a legit F31 diagnosis (called “non-compliant” F31 from now on), prior to the first “real” or “compliant” F31 diagnosis, assigned mostly as additional diagnosis. Related to this latter variable we also measured the time between the first “non-compliant” F31 diagnosis (mostly additional diagnosis) and first “compliant” F31 diagnosis as defined in this study. Principal diagnosis alone was considered in inpatient care, while in outpatient care all diagnoses in patient’s medical history were counted. In sensitivity analysis, we tested the effect of considering only the principal diagnosis in outpatient care as well.

### Statistical analysis

Similar to recent studies of the field [20, 27, 28], this article employs Kaplan-Meier survival analysis and Cox proportional hazards model to examine factors associated with the diagnostic delay in patients with bipolar disorder [29]. The Cox proportional model testing multiple covariates at once is specified as follows:

1 $$ h(x)={h}_0\left(\alpha \right)\exp \left({\beta}^Tx\right) $$

where *h*_0_(*α*) is the baseline hazard function, α is a parameter influencing the baseline value, *exp(β)* is the vector of hazard ratios, and *x* is the vector of the predictor variables. The Cox regression estimates the hazard ratios, exp.(β)s—the values of the respective variables differ by one unit, all other covariates being held constant. Variables with exp.(β) s larger than one are associated with increased hazard; the higher the variable, the higher the hazard of the event, in our case, the probability of being diagnosed early with bipolar disorder for the first time.

Statistical calculations are performed using SPSS (version 22.0). Data are not subject to substantial measurement errors, neither right-censoring nor truncation applies: each patient in the sample was diagnosed with bipolar disorder and the sample design involved no thresholds. A few observations are left-censored; patients may have entered specialist mental healthcare system prior to 1 January 2004. The covariates are entered into the Cox model in one single step. For multiple highly correlated covariates (with coefficients higher than 0.7), only one variable from the set of intercorrelated variables is used [30]. Proportionality of hazards was tested using Schoenfeld residuals [31]. Omnibus tests of model coefficients were conducted for assessing the validity of the model [32]. The omnibus test is a likelihood-ratio chi-square test of the full model versus the null model (all the coefficients are zero).

## Results

### Descriptive statistics of the patient population

The population-based cohort included 8935 patients with bipolar disorder. The mean age of patients at the time of the first bipolar diagnosis was 43.59. In the sample, 41.76% of the patients were males, 58.24% were females. In the study period, 88.80% of the patients contacted psychiatric care for the first time in outpatient settings, while 11.20% in inpatient care. Around two-third (64.90%) of the patients were hospitalized with mental problems prior to the first diagnosis of bipolar disorder. 76.60% of the patients received their first diagnosis with bipolar disorder in outpatient care, while 23.40% in inpatient care. 29.98% of the patients received a “non-compliant” F31 diagnosis prior to the first F31 diagnosis, assigned mostly as additional diagnosis. 2.05 years passed between the first “non-compliant” F31 diagnosis and the first F31 diagnosis as defined in this study.

Detailed clinical characteristics of patients with non-zero diagnostic delay (*N* = 7876) are shown in Table 1. We excluded patients with zero diagnostic delay from this table, since these patients did not have any diagnoses prior to their first F31 diagnosis. Data are reported separately for outpatient and inpatient care. Descriptive statistics for predictor variables reveal that prior to the first diagnosis of bipolar disorder, patients with non-zero diagnostic delay were most frequently diagnosed with anxiety disorder, unipolar depression without psychotic symptoms, and schizophrenia in outpatient care, while the most frequent reasons for hospitalization were schizophrenia, unipolar depression without psychotic symptoms, and poisoning by drugs, medicaments and biological substances and toxic effects of substances chiefly nonmedical as to source such as alcohol, petroleum products, detergents, and pesticides.

**Table 1:** Prior ICD-10 diagnosis of patients with bipolar disorder (*N* = 7876)

Diagnosis	Outpatient care					Inpatient care				
	Prevalence	Number of cases per patient				Prevalence	Number of cases per patient			
		Min	Max	Mean	Std. dev.		Min	Max	Mean	Std. dev.
alcohol misuse/dependence (ICD-10 F10.x)	22.73%	0	71	3.41	11.14	9.31%	0	7	0.25	1.01
illicit drug misuse/dependence (ICD-10 F11-F19.x)	17.41%	0	56	1.87	7.73	4.41%	0	3	0.08	0.40
schizophrenia and related disorders (ICD-10 F20-F29.x)	34.19%	0	181	13.65	34.51	21.23%	0	17	0.93	2.75
anxiety disorder (ICD-10 F40-F43.x)	82.91%	0	129	18.59	25.45	18.05%	0	5	0.32	0.85
specific, mixed and other personality disorders other than borderline personality disorder, and enduring personality changes (ICD-10 F60-F62.x excluding F60.3)	23.58%	0	56	2.50	8.41	4.38%	0	2	0.06	0.29
borderline personality disorder (ICD-10 F60.3)	10.89%	0	35	1.10	4.86	2.20%	0	1	0.02	0.15
psychotic depression (ICD-10 F32.3/F33.2)	6.98%	0	23	0.54	2.90	3.96%	0	2	0.06	0.29
unipolar depression without psychotic symptoms (ICD-10 F32.x/F33.x excluding F32.3/F33.2)	64.59%	0	122	14.33	23.91	23.27%	0	10	0.63	1.65
mood affective disorders other than the ones listed above (ICD-10 F30-F39.x excluding F31-F33.x)	21.44%	0	38	1.72	5.85	7.71%	0	3	0.12	0.47
organic, including symptomatic, mental disorders (ICD-10 F00-F09.x)	15.24%	0	42	1.56	6.05	2.83%	0	2	0.04	0.25
neurotic, stress-related and somatoform disorders (ICD-10 F44-F48.x)	14.18%	0	35	1.32	5.08	1.51%	0	1	0.02	0.12
behavioural syndromes associated with physiological disturbances and physical factors (ICD-10 F50–59.x)	17.78%	0	36	1.35	5.17	0.37%	0	9	0.01	0.18
disorders of adult personality and behaviour other than the ones listed above (ICD F63-F69.x)	2.76%	0	4	0.07	0.47	0.32%	0	4	0.00	0.10
mental and behavioural disorders other than the ones listed above (ICD-10 F70-F99.x)	17.05%	0	66	2.52	9.76	4.60%	0	5	0.11	0.61
poisoning and toxic substances (ICD-10 T36-T65.x)		–	–	–	–	22.26%	0	8	0.51	1.33
intentional self-poisoning by drugs, medicaments and biological substances (ICD-10 X60-X64.x)	6.87%	0	3	0.11	0.44	11.95%	0	4	0.20	0.64
intentional self-poisoning by chemicals and noxious substances and intentional self-harm (ICD-10 X65-X84.x)	5.38%	0	3	0.09	0.42	5.76%	0	2	0.08	0.33
intentional self-harm (ICD-10 X60–84.x)	10.42%	0	5	0.29	0.84	15.34%	0	5.00	0.20	0.74

Prevalence shows the proportion of a patients affected by a medical condition from 1 January 2004 to the date of the first diagnosis with bipolar disorder. Number of cases per patient show how many times patients have been diagnosed with a medical condition prior to the date of the first bipolar diagnosis: minimum, maximum and average values are reported for each condition. In outpatient care all diagnoses were considered, in inpatient care only principal diagnoses were included

### Patient pathways and diagnostic delays

**Fig. 1:**
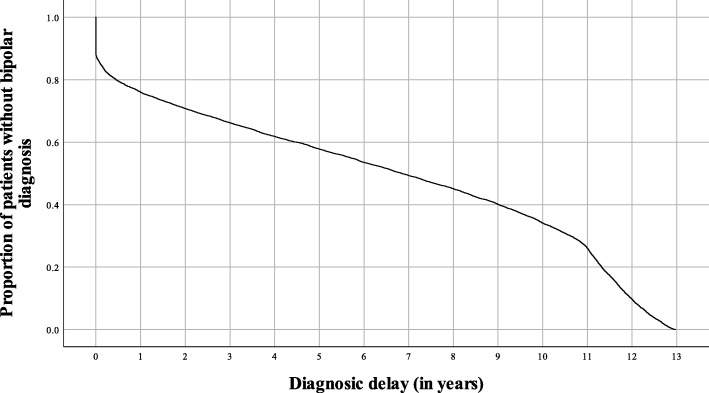
Kaplan-Meier product limit estimates of diagnostic delay**.** The plot shows the relationship between the cumulative proportion of patients without bipolar disorder diagnosis and the time past after entering the specialist mental healthcare system for the first time. Patients with zero diagnostic delay are responsible for the vertical line at the beginning of the survival function. The structural break in the curve at 11 years can be explained by the availability of electronic records; patients being diagnosed with bipolar disorder early 2015 might have a delay of 11 years as maximum, while patients being diagnosed with bipolar disorder late 2016 might have a delay of 13 years as a maximum

In the cohort (*N* = 8935), the time to diagnosis of bipolar disorder measured from the date of the first presentation to any specialist mental healthcare service was 6.46 years on average. The median diagnostic delay was 6.85 years. The diagnostic delay ranged from 0 to 12.98 years, with interquartile range (25th vs 75th percentile) from 1.17 to 11.05 years. There were 1059 (11.85%) patients with zero diagnostic delay; these patients were diagnosed with bipolar disorder on the day of contacting a mental healthcare service for the first time during the sample period of 13 years. The third quartile was closer to the maximum than to the median due to the diagnostic delay being bounded from above. As no electronic records were available for the study before 1 January 2004, several patients might have been admitted to a specialist mental healthcare service earlier than what was deducted from the data. The Kaplan-Meyer plot (Fig. 1) highlights the relationship between the cumulative proportion of patients not being diagnosed with bipolar disorder and time past after having the first entry into the specialist mental healthcare system. Patients with zero diagnostic delay are responsible for the vertical line at the beginning of the survival function. The structural break in the curve at 11 years can be explained by the availability of electronic records.

**Fig. 2:**
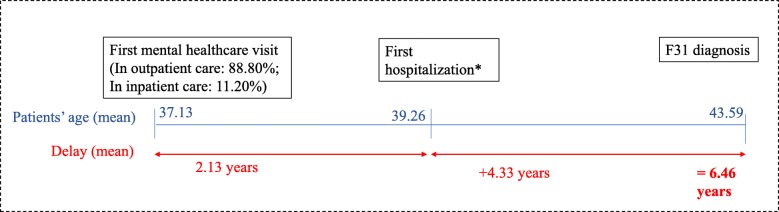
Timeline for patients with bipolar disorder (*N* = 8935). The figure shows the sequential timeline of medical history for all patients in the cohort. Patients entered the specialist mental healthcare system for the first time with a mean age of 37.13 years. Patients were hospitalized 2.13 years later for the first time with a mean age of 39.26 years. Patients received their first F31 diagnosis at a mean age of 43.59; the time to diagnosis of bipolar disorder measured from the date of the first presentation to any specialist mental healthcare service was 6.46 years on average. * Age at first hospitalization is calculated only for patients being hospitalized (*N* = 6608; 63,99%)

In the study period, 88.80% of the patients contacted psychiatric care for the first time in outpatient settings, while 11.20% in inpatient care. Figure 2 shows the sequential timeline of medical history for all patients in the cohort. Nevertheless, there were large variations across patients. For example, 11.85% of patients were diagnosed with bipolar disorder without any delay, and slightly more than one-third of the patients (35.10%) were never hospitalized with mental health problems.

**Fig. 3:**
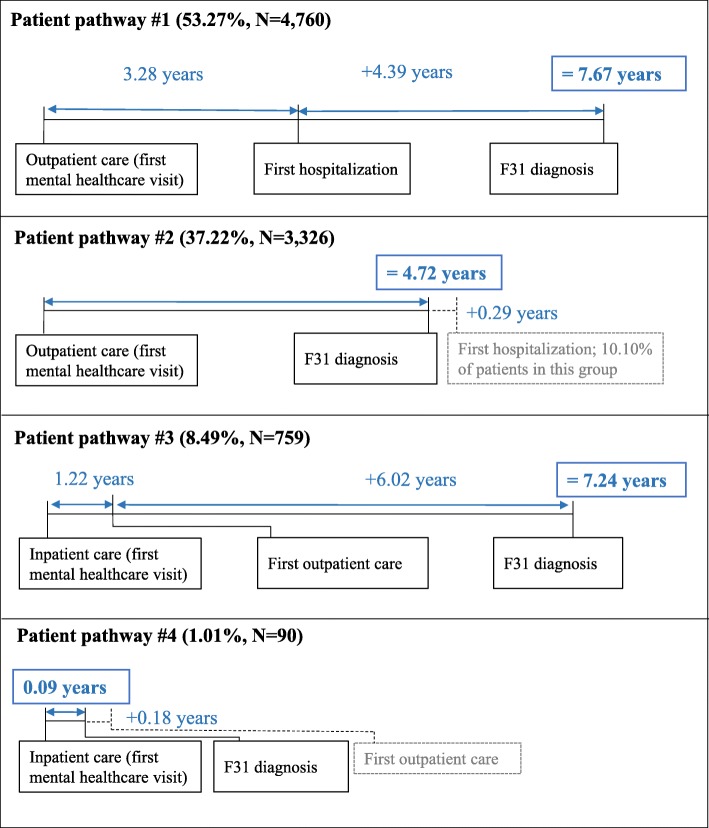
Patient pathways and diagnostic delays. The figure shows four patient pathways. Patient pathway #1 includes patients who entered the mental healthcare system in outpatient care and were later hospitalized with mental healthcare problems. Patient pathway #2 includes patients with outpatient visits only prior to their first F31 diagnosis. Patient pathway #3 encompasses patients who were first hospitalized with mental health problems and later appeared in psychiatric or addictology outpatient care. Patient pathway #4 includes patiehts who were diagnosed with bipolar disorder in inpatient care without ever consulting a specialist in outpatient care

As shown in Fig. 3, there are four patient pathways; the two most common ones characterize more than 90% of patients. In the cohort, more than half of patients (53.27%) consulted first mental healthcare services in outpatient care and was later hospitalized with mental healthcare problems; these patients received their first F31 diagnosis either in outpatient or in inpatient care. The diagnostic delay was the longest in this category. For a typical patient, 3.28 years passed from the first outpatient visit until the first hospitalization with mental health problems. From this hospitalization, an additional 4.39 years passed until the patient was diagnosed with bipolar disorder, resulting in a total delay of 7.67 years. The second typical category comprised 37.22% of patients, whose pathway only included outpatient visits, including the one where they were diagnosed with bipolar disorder. Slightly more than one-tenth of patients (10.31%) in this category were hospitalized after the bipolar diagnosis at least once with mental health problems. The delay in this category was below the average, 4.72 years. Patients in the third, less typical category were first hospitalized with mental health problems, and on average after 1.22 years following the discharge appeared in psychiatric or addictology outpatient care. For a typical patient in this category, 6.02 years passed from this outpatient visit until the first diagnosis of bipolar disorder either in outpatient or in inpatient care, resulting in a total delay of 7.24 years. Patients in the fourth, rather marginal category, were diagnosed with bipolar disorder in inpatient care without ever consulting a specialist in outpatient care. The diagnostic delay was the shortest in this category; patients were assigned with a bipolar disorder diagnosis in 0.09 years on average after the first hospitalization. Most patients in this category (91.11%) received the diagnosis of bipolar disorder during their first inpatient stay, i.e. there was no delay.

### Factors associated with diagnostic delay

There were 1059 (11.85%) patients with no diagnostic delay; these patients were diagnosed with bipolar disorder on the day of showing up at a mental healthcare institution for the first time. As shown in Table 2, men and younger patients have a higher probability to fall into this group. Although close to 89% of patients showed up in outpatient care in both the zero and non-zero delay groups, patients with zero delay were diagnosed with higher probability in inpatient care than patients with non-zero delay. Several of them were referred immediately to a hospital after visiting an outpatient clinic. Only 3.49% of patients with zero-delay had a prior F31 diagnosis from non-specialist mental health professionals, mostly from a neurologist (outpatient care), and from internists or intensive care units (inpatient care). In contrast, 35.97% of patients with non-zero delay had a prior F31 diagnosis, not complying with our inclusion criteria, mostly as secondary diagnosis from psychiatrists and neurologists. The time between this prior, “non-compliant” F31 diagnosis and the first F31 diagnosis as defined in this study (mostly principal diagnosis from psychiatric institutions) was much longer for patients with zero diagnostic delay than for patients with non-zero delay (3.40 vs 1.97 years).

**Table 2:** Characteristics of patients with zero vs non-zero diagnostic delay

		zero diagnostic delay (*N* = 1059)	non-zero diagnostic delay (*N* = 7876)
Gender	female	541 (51.09%)	4663 (59.21%)
	male	518 (48.91%)	3213 (40.79%)
Age categories	18–27	207 (19.55%)	1061 (13.47%)
	28–37	251 (23.70%)	1434 (18.21%)
	38–47	267 (25.21%)	1930 (24.50%)
	48–57	212 (20.02%)	1994 (25.32%)
	58–65	122 (11.52%)	1457 (18.50%)
First consultation with mental healthcare specialists	inpatient care	119 (11.24%)	882 (11.20%)
	outpatient care	940 (88.76%)	6994 (88.80%)
F31 diagnosis	inpatient care	889 (83.95%)	5955 (75.61%)
	outpatient care	170 (16.05%)	1921 (24.39%)
Prior F31 diagnosis, assigned mostly as additional diagnosis (number of patients)		37 (3.49%)	2833 (35.97%)
Time between prior F31 diagnosis (mostly additional diagnosis) and first F31 diagnosis as defined in this study (years)		3.40	1.97

Table 3 shows the factors associated with diagnostic delay in patients with bipolar disorder obtained from a multivariable Cox regression analysis. Patients with no delay were excluded from the model due to the lack of mental health history. In inpatient care, principal diagnosis alone was considered. In outpatient care, in model 1 all diagnoses in patients’ medical history were counted, while in model 2 we tested the effect of considering only the principal diagnosis. In model 1, the categorical variable of age groups revealed the effect of age; the older the patients, the higher the probability of delayed bipolar diagnosis. There was no significant difference in diagnostic delay between male and female patients. Only two clinical predictors were significantly associated with a decreased diagnostic delay at the 5% significance level: 1) the time to diagnosis of bipolar disorder measured from the date of the first presentation to any specialist mental healthcare service was only just shorter, if patients had an earlier “non-compliant” bipolar disorder diagnosis; and 2) the diagnostic delay was also marginally shorter, if patients were diagnosed with neurotic, stress-related and somatoform disorders in outpatient care. In contrast, diagnoses of many other psychiatric disorders received after the first contact were coupled with an increase in the delay. We found empirical evidence that in both outpatient and inpatient care prior diagnoses of schizophrenia, specific, mixed and other personality disorders other than borderline personality disorder, and enduring personality changes, and unipolar depression without psychotic symptoms were associated with increased diagnostic delay. We have to note, however that effect sizes are much smaller in outpatient care. Furthermore, in outpatient care, illicit drug misuse and dependence, prior diagnoses of anxiety disorder, borderline personality disorder and mood affective disorders other than depression were also associated with slightly increased diagnostic delay. In inpatient care, prior diagnoses of alcohol misuse and dependence, neurotic, stress-related and somatoform disorders, and behavioural syndromes associated with physiological disturbances and physical factors were also associated with increased diagnostic delay. Patient pathways were also associated with diagnostic delay; if patients consulted mental healthcare specialists in outpatient care first, the hazard of delayed diagnosis increased considerably. Similarly, the diagnostic delay was longer, if patients were hospitalized. Sensitivity analysis was performed on diagnostic coding in outpatient care. Comparable results were obtained using only the principal diagnoses given in outpatient care (Table 3, model 2).

**Table 3:** Factors associated with diagnostic delay in patients with bipolar disorder (*N* = 7876)

Factors		Number of patients	Percentage	Model 1			Model 2		
				Adjusted hazard ratio	95% confidence interval	Sig.	Adjusted hazard ratio	95% confidence interval	Sig.
Age categories	18–27	1061	13.47%	Reference			Reference		
	28–37	1434	18.21%	0.65	0.60–0.71	0.000 ***	0.63	0.58–0.69	0.000 ***
	38–47	1930	24.50%	0.51	0.47–0.56	0.000 ***	0.50	0.46–0.55	0.000 ***
	48–57	1994	25.32%	0.47	0.43–0.51	0.000 ***	0.46	0.42–0.50	0.000 ***
	58–65	1457	18.50%	0.46	0.42–0.50	0.000 ***	0.44	0.41–0.49	0.000 ***
Gender	Female	4663	59.21%	Reference			Reference		
	Male	3213	40.79%	1.03	0.98–1.08	0.242	1.02	0.97–1.07	0.366
First mental health visit	Inpatient	1157	14.69%	Reference			Reference		
	Outpatient	6719	85.31%	0.62	0.58–0.67	0.000 ***	0.65	0.61–0.70	0.000 ***
First diagnosis with bipolar disorder	Inpatient	5955	75.61%	Reference			Reference		
	Outpatient	1921	24.39%	1.01	0.96–1.07	0.678	1.01	0.95–1.06	0.806
Prior F31 diagnosis, assigned mostly as additional diagnosis (proportion of patients)		2737	34.75%	1.01	1.01–1.01	0.000 ***	1.01	1.01–1.01	0.000 ***
Time between prior F31 diagnosis (mostly additional diagnosis) and first F31 diagnosis as defined in this study (years)		2814	35.73%	1.00	1.00–1.00	0.000 ***	1.00	1.00–1.00	0.000 ***
Patient was hospitalized with mental problems	No	3012	38.24%	Reference			Reference		
	Yes	4864	61.76%	0.92	0.87–0.98	0.006 *	0.91	0.86–0.97	0.002 *
Mania at first presentation	No	2782	35.32%	Reference			Reference		
	Yes	5094	64.68%	0.98	0.93–1.03	0.391	0.96	0.92–1.01	0.132
Inpatient care	alcohol misuse/dependence	733	9.31%	0.93	0.90–0.96	0.000 ***	0.95	0.92–0.98	0.000 ***
	illicit drug misuse/dependence	347	4.41%	0.98	0.92–1.05	0.563	1.01	0.95–1.08	0.721
	schizophrenia and related disorders	1672	21.23%	0.97	0.96–0.98	0.000 ***	0.97	0.96–0.98	0.000 ***
	anxiety disorder	1422	18.05%	0.99	0.97–1.02	0.696	1.01	0.98–1.04	0.498
	specific, mixed and other personality disorders other than borderline personality disorder, and enduring personality changes	345	4.38%	0.90	0.83–0.98	0.014 *	0.95	0.88–1.03	0.248
	borderline personality disorder	173	2.20%	0.88	0.75–1.04	0.128	0.98	0.83–1.16	0.832
	psychotic depression	312	3.96%	1.07	0.98–1.17	0.129	1.13	1.03–1.23	0.008 *
	unipolar depression without psychotic symptoms	1833	23.27%	0.98	0.97–1.00	0.042 *	0.99	0.97–1.00	0.065 †
	mood affective disorders other than the ones listed above	607	7.71%	0.99	0.94–1.04	0.594	1.00	0.95–1.05	0.931
	organic, including symptomatic, mental disorders	223	2.83%	1.02	0.91–1.14	0.706	1.02	0.91–1.14	0.728
	neurotic, stress-related and somatoform disorders	119	1.51%	0.77	0.64–0.94	0.008 *	0.77	0.63–0.93	0.008 *
	behavioural syndromes associated with physiological disturbances and physical factors	29	0.37%	0.81	0.71–0.93	0.003 *	0.82	0.72–0.93	0.002 *
	disorders of adult personality and behavior other than the ones listed above	25	0.32%	1.01	0.82–1.25	0.937	1.01	0.83–1.24	0.903
	mental and behavioural disorders other than the ones listed above	362	4.60%	0.93	0.87–0.99	0.026 *	0.94	0.88–1.01	0.087 †
	poisoning and toxic substances	1753	22.26%	0.98	0.94–1.02	0.366	1.00	0.96–1.04	0.818
	intentional self-harm	1208	15.34%	1.04	0.99–1.09	0.129	1.03	0.98–1.08	0.256
Outpatient care	alcohol misuse/dependence	1790	22.73%	1.00	1.00–1.00	0.088 †	0.99	0.98–0.99	0.000 ***
	illicit drug misuse/dependence	1371	17.41%	1.00	0.99–1.00	0.025 *	0.98	0.98–0.99	0.000 ***
	schizophrenia and related disorders	2693	34.19%	0.99	0.99–1.00	0.000 ***	0.99	0.99–0.99	0.000 ***
	anxiety disorder	6530	82.91%	1.00	0.99–1.00	0.000 ***	0.99	0.99–0.99	0.000 ***
	specific, mixed and other personality disorders other than borderline personality disorder, and enduring personality changes	1857	23.58%	1.00	0.99–1.00	0.005 *	0.97	0.95–0.99	0.005 *
	borderline personality disorder	858	10.89%	0.99	0.99–1.00	0.003 *	0.96	0.94–0.99	0.002 *
	psychotic depression	550	6.98%	1.00	0.99–1.01	0.702	0.98	0.97–0.99	0.006 *
	unipolar depression without psychotic symptoms	5087	64.59%	0.99	0.99–0.99	0.000 ***	0.99	0.99–0.99	0.000 ***
	mood affective disorders other than the ones listed above	1689	21.44%	0.99	0.98–0.99	0.000 ***	0.98	0.97–0.98	0.000 ***
	organic, including symptomatic, mental disorders	1200	15.24%	1.00	0.99–1.00	0.315	0.98	0.97–0.99	0.000 ***
	neurotic, stress-related and somatoform disorders	1117	14.18%	1.01	1.00–1.01	0.001 *	0.97	0.95–0.98	0.000 ***
	behavioural syndromes associated with physiological disturbances and physical factors	1400	17.78%	1.00	0.99–1.00	0.071 †	1.01	0.97–1.05	0.592
	disorders of adult personality and behavior other than the ones listed above	217	2.76%	1.00	0.95–1.06	0.928	1.00	0.99–1.01	0.897
	mental and behavioural disorders other than the ones listed above	1343	17.05%	0.98	0.98–0.99	0.000 ***	0.97	0.96–0.97	0.000 ***
	intentional self-harm	821	10.42%	0.98	0.95–1.02	0.362	0.98	0.94–1.02	0.331

In inpatient care, in both model 1 and model 2 principal diagnosis alone was considered. In outpatient care, in model 1 all diagnoses in patient’s medical history were counted, while in model 2 we tested the effect of considering only the principal diagnosis. Variables with Exp(β) smaller than one are associated with decreased hazard; the lower the variable, the lower the hazard of the event. For example, in model 1 for the variable alcohol misuse/dependence in inpatient care the interpterion of Exp(β) is as follows: the probability of being diagnosed early with bipolar disorder for the first time is by 7% lower (1–0.93 = 0.07) if a patient has received an additional diagnosis of alcohol misuse/dependence in inpatient care

† *p* < 0.1; * *p* < 0.05; *** *p* < 0.0001

Table 3: Factors associated with diagnostic delay in patients with bipolar disorder.

For many clinical predictors, the assumption of proportional hazards did not hold, among others for alcohol misuse/dependence, schizophrenia and related disorders, anxiety disorder, and unipolar depression without psychotic symptoms in both inpatient and outpatient care (Table 4). Large sample size may be responsible for the evidence against the proportionality of hazards [33]. The graphical analysis of the transformed log-minus-log survival curves indicated no major violation of the proportional hazard hypothesis; the transformed curves did not intersect.

**Table 4:** Test of proportionality of hazards using Schoenfeld residuals

Factors		rho	χ^2^	Sig.
Age categories	18–27	Reference		
	28–37	0.010	0.780	0.377
	38–47	0.004	0.120	0.729
	48–57	0.014	1.760	0.185
	58–65	0.033	9.430	0.002 *
Gender	Female	Reference		
	Male	0.012	1.250	0.263
First mental health visit	Inpatient	Reference		
	Outpatient	0.053	22.820	0.000 ***
First diagnosis with bipolar disorder	Inpatient	Reference		
	Outpatient	0.012	1.100	0.294
Prior F31 diagnosis, assigned mostly as additional diagnosis (proportion of patients)		0.062	24.820	0.000 ***
Time between prior F31 diagnosis (mostly additional diagnosis) and first F31 diagnosis as defined in this study (years)		0.092	61.380	0.000 ***
Patient was hospitalized with mental problems	No	Reference		
	Yes	0.009	0.710	0.399
Mania at first presentation	No	Reference		
	Yes	0.012	1.220	0.270
Inpatient care	alcohol misuse/dependence	0.042	14.220	0.000 **
	illicit drug misuse/dependence	0.010	0.710	0.400
	schizophrenia and related disorders	0.045	14.960	0.000 **
	anxiety disorder	0.023	4.270	0.039 *
	specific, mixed and other personality disorders other than borderline personality disorder, and enduring personality changes	0.032	8.050	0.005 *
	borderline personality disorder	0.014	1.500	0.220
	psychotic depression	0.022	3.820	0.051 †
	unipolar depression without psychotic symptoms	0.036	10.160	0.001 *
	mood affective disorders other than the ones listed above	0.011	1.020	0.314
	organic, including symptomatic, mental disorders	0.001	0.010	0.912
	neurotic, stress-related and somatoform disorders	0.006	0.250	0.615
	behavioural syndromes associated with physiological disturbances and physical factors	0.022	3.840	0.050 †
	disorders of adult personality and behavior other than the ones listed above	0.003	0.050	0.831
	mental and behavioural disorders other than the ones listed above	0.030	7.430	0.006 *
	poisoning and toxic substances	0.008	0.510	0.475
	intentional self-harm	0.004	0.110	0.746
Outpatient care	alcohol misuse/dependence	0.035	9.600	0.002 *
	illicit drug misuse/dependence	0.026	5.300	0.021 *
	schizophrenia and related disorders	0.109	87.890	0.000 ***
	anxiety disorder	0.118	109.140	0.000 ***
	specific, mixed and other personality disorders other than borderline personality disorder, and enduring personality changes	0.020	3.420	0.065 †
	borderline personality disorder	0.035	9.610	0.002 *
	psychotic depression	0.017	2.180	0.140
	unipolar depression without psychotic symptoms	0.114	106.590	0.000 ***
	mood affective disorders other than the ones listed above	0.056	25.430	0.000 ***
	organic, including symptomatic, mental disorders	0.017	2.400	0.122
	neurotic, stress-related and somatoform disorders	0.014	1.580	0.208
	behavioural syndromes associated with physiological disturbances and physical factors	0.024	4.570	0.033 *
	disorders of adult personality and behavior other than the ones listed above	0.018	2.830	0.092 †
	mental and behavioural disorders other than the ones listed above	0.097	83.440	0.000 ***
	intentional self-harm	0.010	0.840	0.359

† *p* < 0.1; * *p* < 0.05; ** *p* < 0.001; *** *p* < 0.0001

## Discussion

Regarding *patient demographics*, the overrepresentation of females with bipolar disorder is well documented in the literature [8, 20, 21, 34]. Although previous Hungarian epidemiologic survey results showed a nearly equal female-to-male ratio for bipolar disorder [35], this study based on administrative data found that 41.76% of the patients were males, and 58.24% were females. Much less evidence is available on patients’ age at the time of the first diagnosis. In this population-based cohort, the mean age of patients at the time of the first bipolar diagnosis was 47.67. Patients’ age at the time of the bipolar disorder diagnoses is reported to be over 40 years for both Swedish and Danish patients in nationwide bipolar disorder cohorts (45.5 years in 2006 and 40.3 years in 2009 in Sweden, 54.5 years in 1996 and 42.4 years in 2012 in Denmark) [7, 21]. In the interview-based literature, however, patients are reported to be significantly younger at the time of diagnosis. For example, Berk et al. [36] report that patients (*n* = 216) first received a diagnosis of bipolar or schizoaffective disorder at a median age of 30 years. Similarly, Drancourt et al. [8] find that patients (*n* = 501) receive the first mood stabilizer treatment at the age of 34.9 years, keeping in mind that the first drug treatment normally occurs earlier than the final diagnosis [20]. A retrospective study based on self-report is evidently at risk of recall and social desirability biases—rather than what actually occurs in practice, surveys and interviews may simply capture normative responses and expressed attitudes. Although recall bias might contribute to reporting early age diagnosis, it is unlikely to be the only reason for such a large difference. Sample selection bias leading to the overrepresentation of younger patients might provide an additional explanation. Different forms of bipolar disorder might play a crucial role in explaining the variation as well. Kennedy et al. [16] identified early and later onset subgroups while studying the incidence and distribution of first-episode mania by age. They found that the incidence of mania generally peaks in early adult life and has a smaller peak between 40 to 55 years-of-age. It may well be the case that this mid-life peak is more pronounced in Hungary. An alternative explanation is that only minor depressive episodes occur in younger ages that are recognized by general practitioners *as “mere” anxiety, and thus they* mostly prescribe anxiolytics to patients. These patients then, do not appear at the mental health specialist care.

The reported frequencies for diagnoses received from mental healthcare professionals prior to the bipolar disorder diagnosis are in line with the population-wide findings of Carlborg et al. [21] and the etiology of bipolar disorder. The authors report that the most common diagnoses within 4 years prior to the bipolar diagnoses were depressive disorder, depressive recurrent disorder and anxiety—being among the most frequent diagnoses in both outpatient and inpatient care in this study as well. Similarly, the reported frequencies are in agreement with the findings of Patel et al. [20] as well. The authors report that schizophrenia or related disorders, unipolar depression without psychotic symptoms, and anxiety disorder are the most common prior diagnoses.

In a large cohort, we investigated the delayuntil the diagnosis of bipolar disorder from the first admission to outpatient or inpatient specialist mental care settings. The mean *diagnostic delay* was 6.46 years but varied significantly across patients with an interquartile range of 1.17–11.05 years. This finding is in line with the interview-based estimate of Berk et al. [36] and the nationwide registry-based calculation of Carlborg et al. [21] and Medici et al. [7]; in the first study, the authors report that patients with bipolar or schizoaffective disorder first sought medical treatment at 24 years and first received a diagnosis of bipolar or schizoaffective disorder at 30 years, resulting in a delay of 6 years, while in the latter two studies the authors found that mean time from the first psychiatric diagnosis to the bipolar diagnosis is 6.23 years in Sweden and 7.9 years in Denmark. It is important to note that normally substantial delays are encountered from first symptoms to seeking medical treatment; Berk et al. [36] find that 6.5 years pass from the first symptoms to the first consultation with a mental healthcare specialist, resulting in a total delay of 12.5 years from the onset of mental illness. Most other studies report shorter delays, however. Hirschfeld et al. [5], for example, document a delay of 10 years between the first symptom and the final diagnosis of bipolar disorder; while Drancourt et al. [8] find the delay in treatment from the illness onset to be 9.6 years. A recent meta-analysis covering 51 samples characterized by high between-sample heterogeneity concluded that the interval between the onset of bipolar disorder and its management is 5.8 years [9]. Management was defined as assigning the diagnosis of bipolar disorder (rather than first hospitalization or first treatment). This meta-analytical delay estimate is shorter than the one suggested by this study: 5.8 years from onset until management versus 6.44 years from seeking medical treatment to diagnosis. Dagani et al. [9] found that increased reported delay between the onset and the initial management was associated with three factors: 1) onset was defined as the first episode (rather than onset of illness or symptoms); 2) the study was more recent; and 3) the study employed a systematic method for detecting the chronology of illness. All but the first aspect holds for our current study, partly explaining the relatively high delay. Dagani et al. [9] hypothesized that the counterintuitive result of longer delay with onset defined as the first episode is due to the fact that the onset of symptoms refers to the manic episode rather than the symptoms of depression. Sample size might provide an additional explanation for the difference; the sample size is much larger in this study than in any previous work. The meta-analysis pooled information from 27 studies on 9415 patients together. The population size in this study is almost three times larger than the largest sample of 3536 hospitalized patients and is slightly larger than the entire pooled sample. In addition to the large sample size, data from both inpatient and outpatient mental healthcare care were collected, and the electronic health records, being exempt from recall bias, covered an exceptionally long period (11 years for patients receiving the first bipolar disorder diagnosis early 2015 and 13 years for patient being diagnosed with bipolar disorder late 2016).

When we measured the diagnostic delay for patient groups with different pathways, we found the delay to be the longest for patients whose care were shared between outpatient and inpatient providers, regardless whether patients visited a mental healthcare institution in outpatient or inpatient setting first (7.67 and 7.24 years, respectively; see Fig. 3). In contrast, if patients were treated in outpatient or inpatient care only, the diagnostic delay was much shorter (4.72 and 0.09 years, respectively). Vast empirical evidence suggests that there are significant deficits in communication and information transfer between outpatient and inpatient-based physicians which adversely affects patient care [37–39]. Greater continuity of care might thus be associated not only with better health outcomes, but also with earlier diagnosis. To the authors’ knowledge, although this association has been recorded in many other domains [40–43], it has never been documented for patients with mental disorders. Due to the endogeneity of case severity, this relationship is mere speculation as yet. Carefully designed future research should investigate this possible linkage further.

It was reasonable to assume that if, at the time of the bipolar diagnosis, the sub-diagnosis was any kind of mania then the delay is shorter due to the straightforward nature of manic episodes. Nevertheless, this variable turned out to be insignificant.

Results of the Cox analysis showed that the older the patient at the time of the first bipolar diagnosis, the longer the delay. This can be explained by physicians having a higher probability of associating symptoms of bipolarity to bipolarity at younger ages, when the disorder is more likely to occur. Given the higher than expected average age of patients at the time of the first bipolar disorder diagnosis in our study population as compared to previous literature [7, 8, 21, 36], it is conceivable that the first show-up also happens at later ages. This in turn, together with the fact that progressed age elongate delay, might also explain why we found a somewhat longer delay than other similar studies.

We also found that first mental health visit in outpatient setting as opposed to inpatient setting increased delay. This phenomenon presumably reflects the fact that the less severe cases are more prone to show up in outpatient clinics which makes straightforward diagnosis more difficult. Also, these patients usually have less indicative-of-bipolarity events in their past that may point toward bipolarity. Interestingly enough, independent of the place of the first visit, hospitalization during the course of the patient pathway itself had a detrimental effect on delay. This is rather counterintuitive, precisely because of the arguments laid out above on how the first visit in an outpatient setting augments delay.

Among comorbidities diagnosed prior to the diagnosis of bipolar disorder, neurotic, stress-related and somatoform disorders and behavioural syndromes associated with physiological disturbances and physical factors considerably elevates the risk of delay when given in an inpatient setting. Moderately increase delay, if patients were given the diagnosis of alcohol misuse, specific, mixed and other personality disorders, other mental and behavioural disorders and intentional self-poisoning by drugs in a hospital. Furthermore, schizophrenia, depression without psychotic symptoms at any setting and drug misuse, anxiety disorder, other mood affective disorders, specific, mixed and other personality disorders, borderline personality disorder, other mood affective disorders, and other mental and behavioural disorders, all given at outpatient settings, add slightly to the delay. We assume that all of these conditions complicate the matter at hand, thus making the diagnosis harder to set up. It is interesting to note, however, that neurotic, stress-related and somatoform disorders have opposing effects on delay in outpatient and inpatient settings. The cause of this phenomenon is not clear.

Although our sensitivity analysis – using only principal diagnoses in outpatient cases - lead to very similar results, there are notable differences in the significance of some explanatory variables. For example, in the case when we work with only principal outpatient diagnoses, psychotic depression given at hospitals is significant, with a non-negligible reduction in diagnostic delay. A possible explanation is that manic episodes follow this severe state more often and more early than other, less serious conditions, and manic episodes coupled with psychotic depression make the case for bipolarity. It is hard to explain, however, why psychotic depression in outpatient settings have a reverse, nevertheless much smaller, effect (still considering principal diagnoses only). Another substantial difference in the sensitivity analysis that specific, mixed and other personality disorder and borderline personality disorder given in outpatient settings, have 5–6 times larger effect on delay, albeit still moderate in absolute numbers. The regression coefficient of neurotic, stress-related and somatoform disorders, at the same time, changes sign and have 4 times larger effect size. The changed direction of the effect – i.e. increasing delay – is more in line with the effects of this comorbidity in the hospital setting, but we have no clear view, why is the baseline direction counterintuitive.

There was an additional hypothesis considering variables affecting diagnostic delay that we tested in models but omitted from the final regression due to a lack of significance. Since the hypothesis was based on sound theoretical background, we felt useful to report it here. The idea was that men, who got a previous diagnosis of alcohol-related disorders will have longer delay. This is a common comorbidity of bipolar males in Hungary that might mask the underlying bipolarity, thus leading to an elevated delay. This dummy variable turned out to be insignificant.

### Strengths and limitations

Our findings might be generalizable in a variety of settings. The data used in this research are free from information and recall bias—they were retrieved from electronic health records. Moreover, a large population-based cohort constitutes the sample—the data is free from selection bias. The sample is drawn from a population of close to 10 000 000 individuals.

This study has several limitations, too. First of all, we implicitly assumed that patients at the time of the first presentation to any specialist mental healthcare institution have already experienced the onset of bipolar disorder. In this, we followed the approach of Patel et al. [20], thus conforming with already accepted criteria. Considering the more than 10 years of difference between the age at onset of bipolar disorder estimated in other studies and the mean age of patients at the time of the first presentation to any specialist mental healthcare institution in this study (20–25 years vs 35.7 years), the majority of the patients in the sample most probably have already experienced the onset of bipolar disorder at the time of the first presentation [8, 23, 24]. Nevertheless, we cannot rule out the possibility that the delay for some patients is shorter as they experience the first symptoms of bipolarity later. Second, we have to emphasize, that a part of the diagnostic delay is normal, in the sense that for the majority of cases, the nature of bipolar disorder does not allow for clinicians to immediately assign the diagnosis of bipolarity, but rather they have to perform further clinical observations. Inherent causes of diagnostic delay are numerous. One is that the only way to assign bipolarity diagnosis at the very first presentation is when the patient is having a manic episode. All the other symptoms are non-specific to bipolar disorder. Another is that doctors are reluctant to rush into the diagnosis of bipolarity before it is rock solid due to the stigmatization of these patients. Moreover, approximately half of mental health patients have more than one mental disorder. If a particular symptom precedes bipolar symptoms this can either be because of an unrelated comorbidity, or a precursor to bipolar disorder or a disease that advanced the onset of bipolarity. It is impossible, however, to disentangle the above three cases in hindsight. Third, we investigated factors associated with diagnostic delay from the date of the first presentation to any specialist mental healthcare institutions. However, some patients may have initially presented to general practitioners. Minor depressive episodes may typically be handled by general practitioners, mostly by prescribing anxiolytics to patients. These patients either do not appear at the mental health specialist care or present distortions in the time of the first presentation at the health care system with mental disorders. This approach also inhibited us to use primary care-related patient pathway variables. Further research examining administrative data from primary care may address this limitation. Fourth, electronic records from specialist mental care were available from 1 January 2004 only. Although the medical history of over 10 years can be considered as fairly long, the diagnostic delay had an upper limit of 13 years in this study. In reality, some patients might have been admitted to specialist mental healthcare service earlier than what was deducted from the data; the diagnostic delay for these patients is longer than the one estimated in this study. Thus, the average delay of 6.46 years shall be considered as a lower-bound estimate. Fifth, as we retrieved data from an administrative financing database diagnosis misclassification due to economic or other incentives may bias study findings. For example, the shorter delay in patients diagnosed with somatoform disorder in outpatient care may partially reflect the psychiatrists’ desire to use mood stabilizers for the treatment of somatization, and therefore over-diagnose the patient as having bipolar disorder. Sixth, for many clinical predictors, the assumption of proportional hazards did not hold. Although the differences in the direction of hazard ratios between predictor variables provided meaningful conclusions, the relative magnitude of hazards was not comparable. Seventh, several other factors might influence diagnostic delay, among others, marital, social and socioeconomic status of the patient, and the clinical experience of the mental healthcare professionals who first assess patients—data not recorded routinely in administrative databases. Eighth, patient pathways were captured by basic indicators. Future research shall address how the temporal sequence of mental healthcare institutions and caring doctors are associated with diagnostic delay. Finally, assessing the length and the factors associated with treatment delay were beyond the scope of this study. As documented in the literature, treatment delay is shorter than diagnostic delay reflecting the initiation of treatment prior to assigning a formal diagnosis of bipolar disorder [20].

## Conclusions

Our large sample of more than 8000 bipolar patients, from administrative databases allowing for a 13-year study period, shed light on the difficulties with diagnosing bipolarity in Hungary. We found that the mean age of patients at the time of the first bipolar diagnosis was 43.59 years, while the diagnostic delay was 6.46 years on average. Our results are comparable with studies conducted in Denmark and Sweden, using administrative data [7–21]. In the sample, 1.85% of patients were diagnosed with bipolar disorder without any delay, and slightly more than one-third of the patients (35.10%) were never hospitalized with mental health problems. 88.80% of the patients contacted psychiatric care for the first time in outpatient settings, while 11% in inpatient care. Diagnostic delay was shorter, if patients were diagnosed with bipolar disorder by non-specialist mental health professionals before. In contrast, diagnoses of many psychiatric disorders received after the first contact were coupled with delayed bipolar diagnosis. We found empirical evidence that in both outpatient and inpatient care prior diagnoses of schizophrenia, unipolar depression without psychotic symptoms, and several disorders of adult personality were associated with increased diagnostic delay. Patient pathways played an important role as well; the hazard of delayed diagnosis increased if patients consulted mental healthcare specialists in outpatient care first or they were hospitalized.

This study is a first attempt to systematically describe and analyse the diagnosis of bipolar patients in Hungary controlling for possible confounders. Our focus was more on clinical variables as opposed to factors controllable by policy-makers. This is in line with the current state of accumulated knowledge on bipolar diagnostic delay. We currently are unable to disentangle the “natural” part of the delay, i.e. the part that is due to the normal course of the disease and the proper application of clinical diagnostic criteria. In theory, it would be possible to determine this “natural” part by comparing results from different countries, but most likely there would be context-specific issues distorting this comparison. Since we do not have information yet on these country-specific causal structures relating to the delay, it is too early to formulate relevant policy recommendations. Further analysis of zero-delay patients, non-compliant diagnoses of bipolarity or how care continuity affects the delay, however, could illuminate care characteristics that have implications for health policy.

## Data Availability

The approval to access the anonymized data maintained by the National Healthcare Service Centre of Hungary (AEEK/4538/2016) requires the data to be treated as confidential and stored on the personal computers of the authorized researchers, protected with multiple passwords. For this reason, the data cannot be shared publicly. However, subject to approval from the directorate general, data access for research purposes is possible by contacting the National Healthcare Service Centre (aeek@aeek.hu) directly.

## Abbreviations

AEEK: National Healthcare Services Centre of Hungary
NHIFA: National Health Insurance Fund Administration of Hungary
